# The prognostic index of m^7^G-related genes in CRC correlates with immune infiltration

**DOI:** 10.1038/s41598-022-25823-w

**Published:** 2022-12-08

**Authors:** Xinkun Huang, Bin Zhu, Chenyu Qian, Ying Feng

**Affiliations:** 1 Department of Gastrointestinal Surgery, Affiliated Hospital of Nantong University, 20 Xisi Street, Nantong, 226001 Jiangsu China; 2 Graduate School of Dalian Medical University, No.9 West Section of South Lushun Road, Dalian, 116000 Liaoning China; 3 Department of General Surgery, Yancheng First Hospital, Affiliated Hospital of Nanjing University Medical School, Yancheng, China; 4 Department of Central Laboratory, Yancheng Medical Research Center of Nanjing University Medical School, Yancheng, China; 5 Medical School, Nantong University, 19 Qixiu Road, Nantong, 226001 Jiangsu China; 6 Research Center of Clinical Medicine, Affiliated Hospital of Nantong University, 20 Xisi Street, Nantong, 226001 Jiangsu China

**Keywords:** Cancer, Immunology, Medical research

## Abstract

*N*7-methyladenosine (m^7^G) modifications have been the subject of growing research interest with respect to their relationship with the progression and treatment of various cancers. This analysis was designed to examine the association between m^7^G-related gene expression and colorectal cancer (CRC) patient outcomes. Initial training analyses were performed using the TCGA dataset, with the GSE28722 dataset then being used to validate these results. Univariate Cox analyses were initially conducted to screen out prognostic m^7^G-related genes, after which a LASSO approach was used to construct an m^7^G risk score (MRS) model. Kaplan–Meier curves, ROC curves, and Cox analyses were subsequently used to validate the prognostic utility of this model in CRC patients. The R maftools package was further employed to assess mutational characteristics in CRC patients in different MRS subgroups, while the ESTIMATE, CIBERSORT, and ssGSEA tools were used to conduct immune infiltration analyses. A WGCNA was then performed to identify key immune-associated hub genes. The EIF4E3, GEMIN5, and NCBP2 genes were used to establish the MRS model. Patients with high MRS scores exhibited worse overall survival than patients with low scores. In Cox analyses, MRS scores were independently associated with CRC patient prognosis. Patients with low MRS scores exhibited a higher tumor mutational burden and higher levels of microsatellite instability. In immune infiltration analyses, higher immune checkpoint expression and greater immune cell infiltration were also observed in patients with low MRS scores. WGCNA analyses further identified 25 CD8+ T cell infiltration-associated genes. These findings suggest that MRS values represent a useful biomarker capable of differentiating among CRC patients with different immunological features and prognostic outcomes, offering an opportunity to better determine which patients are likely to benefit from immune checkpoint inhibitor treatment.

## Introduction

Colorectal cancer (CRC) is among the most common malignancies and was the second leading cause of cancer-associated death in 2020^1^. Primary prevention is one of the main strategies used in an attempt to reduce the rising global prevalence of CRC cases. While colonoscopy procedures are invaluable in this context, they are expensive, necessitate trained endoscopists, and require patient compliance in order to accurately diagnose and treat CRC. When patients with early-stage disease undergo standardized treatment, their 5-year survival rates can exceed 90%^2,3^. However, roughly 30% of patients with CRC already harbor metastases when initially diagnosed, and the 5-year survival rate for these patients is just 20% even with surgical tumor resection and standardized systemic adjuvant therapy^4,5^.

The main treatment strategy for CRC still primarily consists of radical surgical resection together with targeted radiotherapeutic and chemotherapeutic interventions selected based on the condition of a given patient. Curative options for metastatic CRC patients, however, are lacking in most cases. The most frequently applied chemotherapeutic regimen in metastatic CRC cases consists of fluorouracil combined with folinic acid and irinotecan (FOLFIRI), but the emergence of chemoresistance has largely hampered the long-term efficacy of such interventions. Recent advances in molecular biological techniques have enabled the more detailed analysis of the specific genetic and biomolecular factors that drive oncogenic transformation and progression, providing the opportunity to better define diagnostic or prognostic biomarkers for CRC. Treatment with the epidermal growth factor receptor inhibitor cetuximab has led to prolonged CRC patient survival^6^, as has Bevacizumab treatment^7,8^. However, only a limited subset of CRC patients are positioned to benefit from these specialized therapeutic drugs. Unlike conventional therapeutics, immune checkpoint inhibitor (ICI) therapies that target PD-1, PD-L1, and CTLA4 have achieved positive outcomes in many CRC patient subgroups^9–11^. Even so, the benefits of ICI treatment in CRC are often limited, and the efficacy of these ICIs is often influenced by the tumor microenvironment (TME), with few biomarkers currently available that can reliably predict ICI treatment outcomes^12^. Efforts to better define prognostic biomarkers associated with the therapeutic efficacy of different treatment regimens are warranted to better guide the individualized immunotherapeutic treatment of CRC patients.

Over 170 chemical modifications of RNA molecules have been detected to date and shown to influence a diverse range of cellular processes^13,14^. The N7-methylguanosine (m^7^G) modification has been detected on tRNAs, rRNAs, mRNA 5′ caps, and internal regions of RNA molecules, thereby influencing virtually all aspects of mRNA metabolism^15,16^. Notably, recent work suggests that these m^7^G modifications are also related to the onset and progression of various cancers. For example, METTL1 has been reported to increase cisplatin sensitivity in CRC cells through the downregulation of S100 calcium-binding protein A4 (S100A4)^17^. Moreover, a positive correlation between the expression of METTL1 and both advanced clinical stage and high tumor grade has been observed in bladder cancer^18^. The expression of both WDR4 and METTL1 has also been reported to be increased in patients with esophageal squamous cell carcinoma and linked to poorer patient outcomes^19^. In glioma, METTL1 can drive MAPK pathway signaling to enhance tumor growth and proliferation^20^, with WBSCR22 similarly promoting glioma progression^21^. While m^7^G-associated genes are also likely to influence the progression of CRC, their roles in this oncogenic setting have yet to be defined.

In this study, a prognostic biosignature was developed for CRC patients based on m^7^G-associated gene expression. Initially, 29 m^7^G-associated genes were identified based on the GOMF_M7G_5_PPPN_DIPHOSPHATASE_ACTIVITY dataset, the GOMF_RNA_7_METHYLGUANOSINE_CAP_BINDING dataset, the GOMF_ RNA_CAP_BINDING dataset, and a recent review^22^. The expression and mutational profiles for these 29 m^7^G-associated genes were then analyzed among CRC patients in The Cancer Genome Atlas (TCGA), which a LASSO regression model then being constructed based on m^7^G-associated genes that were significant in initial univariate prognostic analyses, leading to the establishment of an m^7^G-related gene score (MRS) model. The predictive utility of this MRS model as a guide to assessing CRC patient prognosis was then validated through Kaplan–Meier, ROC, and Cox analyses and by constructing appropriate nomograms. Genes differentially expressed in different MRS patient subgroups were then subjected to analyses of GO term enrichment, mutational landscapes, and immune cell infiltration. Lastly, key immune infiltration-related hub genes in these MRS-patient subgroups were identified through a weighted gene co-expression network analysis (WGCNA) approach. Overall, these analyses revealed that the developed MRS model offers utility as a prognostic biomarker, with patients in the low-MRS subgroup exhibiting better immune activity such that they were predicted to exhibit better responses to immunotherapeutic treatment.

## Results

### Analyses of CRC patient m^7^G-associated gene expression and mutational profiles

**Figure 1:**
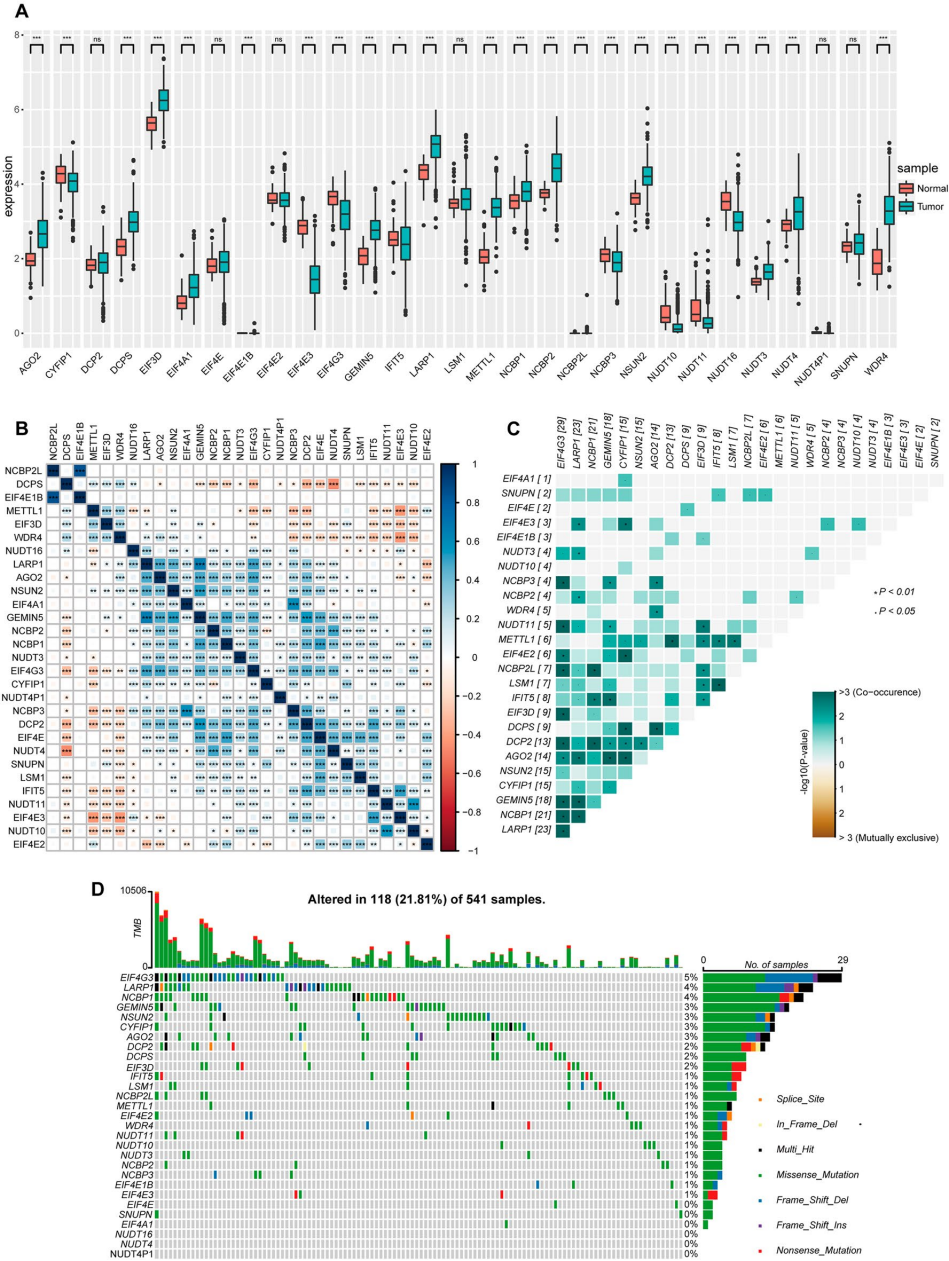
CRC patient m^7^G-related genes expression and mutational profiles. (**A**) The expression of 29 m^7^G-associated genes in normal and CRC patient tumor tissue samples. (**B**) Correlation heat map of expression between m^7^g genes, plotted using the corrplot function in the corrplot package of the R language (version 4.1.2, https://www.r-project.org/). (**C**) The co-occurrence and mutual exclusivity of these 29 m^7^g-related genes after mutation were plotted using the somaticinteraction function in the maftools package of the R language (version 4.1.2). (**D**) Waterfall plots representing the mutational characteristics of these 29 m^7^g-related genes were drawn using the oncoplot function in the maftools package of the R language (version 4.1.2). *P < 0.05; **P < 0.01; ***P < 0.001.

Initially, the expression of 29 different m^7^G-associated genes was analyzed, revealing AGO2, DCPS, EIF3D, EIF4A1, EIF4E1B, GEMIN5, LARP1, METTL1, NCBP1, NCBP2, NCBP2L, NSUN2, NUDT3, NUDT4, and WDR4 to be upregulated in CRC patient tumor tissues, whereas CYFIP1, EIF4E3, EIF4G3, IFIT5, NCBP3, NUDT10, NUDT11, and NUDT16 were downregulated in these tissues (Fig. 1A). In correlation heat maps, some of these genes exhibited co-occurrence whereas others exhibited mutually exclusive expression patterns (Fig. 1B,C). In total, 23 of these 29 genes were found to be mutated in the analyzed CRC patient tumor tissue samples (Fig. 1D), although these mutations were not significantly related with patient OS (Supplementary Fig. 1A).

### MRS model construction

**Figure 2:**
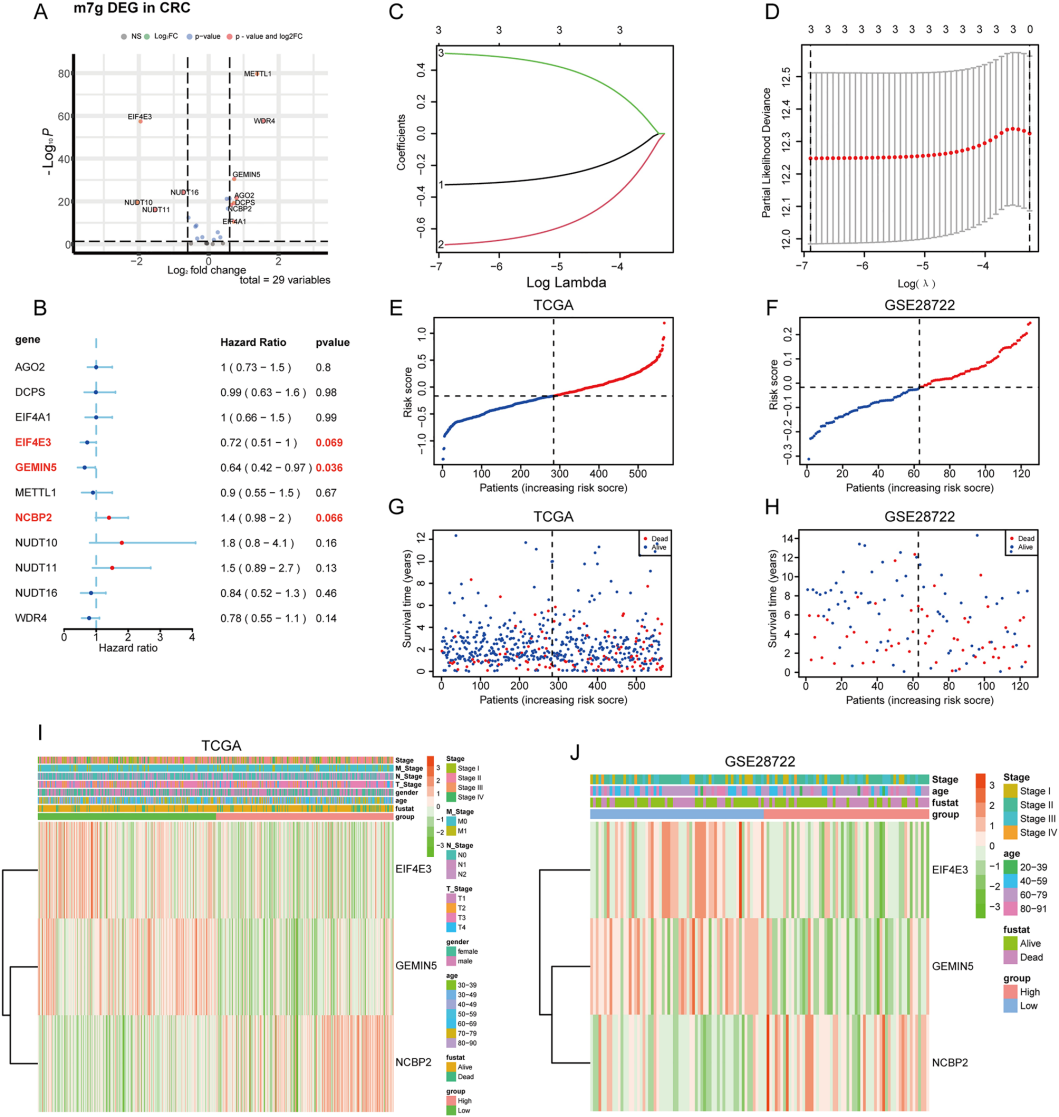
MRS model construction. (**A**) The volcano map depicts 29 identified m^7^g-related genes visualized by EnhancedVolcano package in R language (version 4.1.2). (**B**) Selection of m^7^G-associated genes exhibiting significance in univariate Cox analyses (|logFC|> 0.6, adj.P < 0.1). (**C**) Numbers and coefficient values for different λ values during model construction. (**D**) Continuous adjustment of potential model parameters ultimately yielded the best model (left dashed line) and the simplest model (right dashed line), with the above values corresponding to the number of model features at the indicated λ value. (**E**,**F**) Risk score distributions based on the m^7^G-associated gene model in the training and validation datasets. (**G**,**H**) Differences in survival outcomes between patients in the high- and low-MRS subgroups in the training and validation datasets. (**I**,**J**) Heatmap represents the expression levels of three selected m^7^g-related genes in individual patients, plotted using the pheatmap function in the pheatmap package of the R language (version 4.1.2).

A volcano plot was used to graph these m^7^G-associated genes, with the 11 genes meeting the established significance criteria (|logFC|> 0.6, adj. P < 0.05) being selected for further analysis (Fig. 2A). Of these genes, three exhibited a P-value < 0.1 in univariate Cox analyses (Fig. 2B). Using the TCGA database was used as a training dataset and GSE28722 as a validation dataset, the MRS model was then established as follows through a LASSO regression analysis: MRS = (− 0.322311542390686) × EIF4E3 + (− 0.700547576450906) × GEMIN5 + 0.506142039510906 × NCBP2 (Fig. 2C,D). Risk curves demonstrated that patient risk rises as MRS scores increase (Fig. 2E,F), and the survival of patients in the low- and high-MRS subgroups were analyzed (Fig. 2G,H). Heatmaps depicting the expression of EIF4E3, GEMIN5, and NCBP2 in CRC patients are presented in Fig. 2I,J.

### MRS model validation

**Figure 3:**
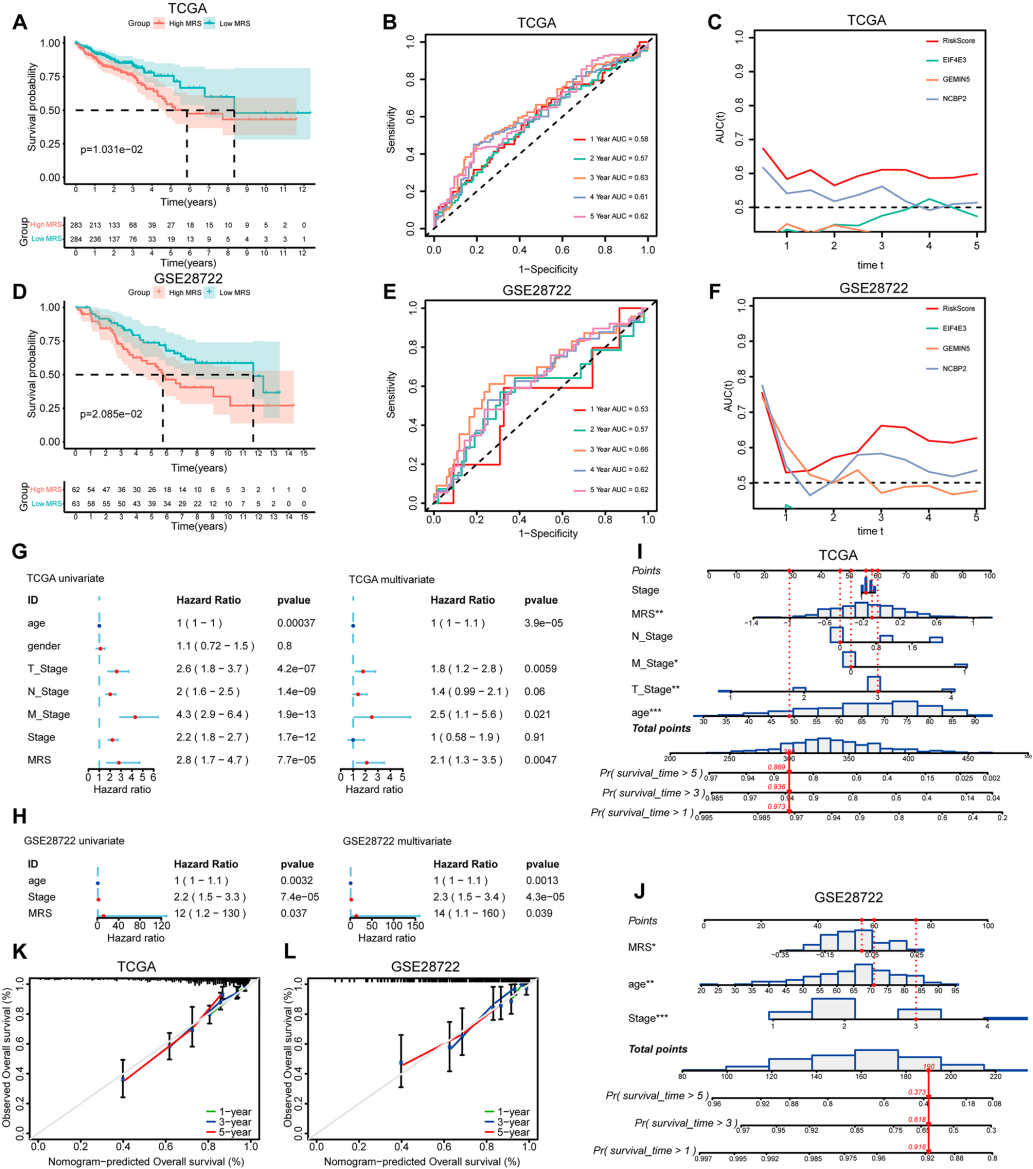
MRS model validation. (**A**–**C**) The training dataset was analyzed using Kaplan–Meier (**A**), ROC (**B**), and Time-ROC curves (**C**). (**D**–**F**) The validation dataset was analyzed using Kaplan–Meier (**D**), ROC (**E**), and Time–ROC curves (**F**). Univariate and multivariate Cox regression analyses were performed for MRS values in the training (**G**) and validation datasets (**H**). An MRS-based nomogram was constructed for the training (**I**) and validation datasets (**J**), with corresponding 1-, 3-, and 5-year calibration curves for these nomograms in the training (**K**) and validation datasets (**L**). *P < 0.05; **P < 0.01; ***P < 0.001.

Those patients in the training and validation datasets exhibiting high MRS scores presented with worse OS than patients in the low-MRS subgroup in Kaplan–Meier analyses (Fig. 3A,D). The area under the ROC curve for this MRS score at 3, 4, and 5 years was > 0.6, consistent with the ability of this model to effectively predict patient prognosis (Fig. 3B,E). This model was also able to predict CRC patient outcomes in a time-ROC analysis (Fig. 3C,F), and univariate and multivariate Cox analyses confirmed that MRS scores were independently associated with CRC patient prognosis (Fig. 3G,H). Predictive nomogram models were further constructed to assess the utility of MRS as a means of predicting the 1-, 3-, and 5-year prognosis of CRC patients (Fig. 3I,J), with calibration curves demonstrating that these MRS scores offered good prognostic utility for all three of these time intervals (Fig. 3K,L).

### The mutational profiles of patients in different MRS subgroups

**Figure 4:**
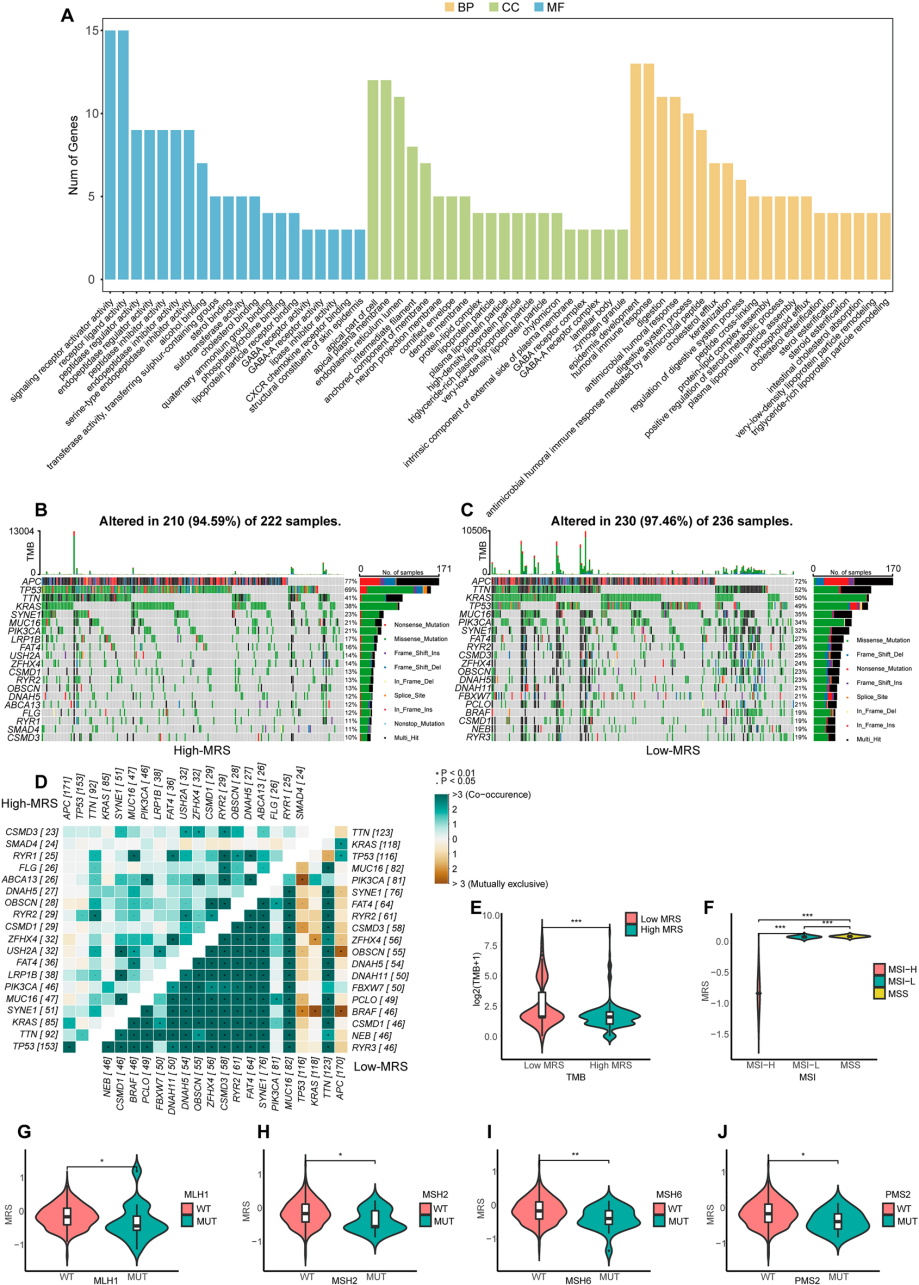
Mutational profiles in different MRS patient subgroups. (**A**) Enrichment analyses for genes differentially expressed in the different MRS patient subgroups. (**B**,**C**) The top 20 genes exhibiting the highest mutational frequencies in patients with high (**B**) and low MRS scores (**C**) were visualized by the maftools package for R language (version 4.1.2). (**D**) The co-occurrence and mutual exclusivity of the genes with the top 20 mutational frequency values in the high- and low-MRS subgroups, were plotted using the somaticinteraction function in the maftools package of the R language (version 4.1.2). (**E**) The association between TMB and MRS subgroups. (**F**) The association between MSI and MRS subgroups. (**G**–**J**) The association between MRS subgroups and the mutational status of the MLH1 (**G**), MSH2 (**H**), MSH6 (**I**), and PMS2 (**J**) genes. *P < 0.05; **P < 0.01; ***P < 0.001.

Next, differential gene expression analyses were conducted by comparing the high- and low-MRS subgroups in the TCGA training dataset, with significant DEGs (|logFC|> 1, adj.P < 0.05) being retained for GO analyses. These genes were significantly enriched in the CXCR chemokine receptor binding, humoral immune response, and other immune pathways (Fig. 4A). Analyses of gene mutations in CRC patients in the TCGA database were then conducted, with the top 20 genes exhibiting the highest mutation frequencies in the high-MRS and low-MRS subgroups being represented with waterfall plots. In the high-MRS subgroup just 7 genes had mutational frequencies > 20% as compared to 16 genes in the low-MRS subgroup. The APC and TP53 genes were less frequently mutated in the low-MRS subgroup relative to the high-MRS subgroup (Fig. 4B,C). CO-occurrence and mutual exclusivity analyses revealed that APC, TP53, and KRAS presented with mutually exclusive relationships with other genes in the low-MRS group, whereas the 17 other genes strongly co-occurred with one another (Fig. 4D). Both tumor mutational burden (TMB) and microsatellite instability (MSI) are important biomarkers that can predict the efficacy of immunotherapeutic interventions. Relative to patients in the high-MRS subgroup, those in the low-MRS subgroup exhibited significantly higher levels of TMB and MSI, with the average MRS score of patients in the MSI-H group consistently being lower than that of patients in the MSI-L and MSS groups (Fig. 4E,F). Mismatch repair (MMR) activity can also be analyzed to gain insight into the repair of errors in DNA replication in tumor cells, with the impairment of this process contributing to higher rates of somatic mutation. The association between mutations in four MMR-related genes (MLH1, MSH2, MSH6, and PMS2) and MRS patient subgroups was analyzed (Fig. 4G–J).

### The immunological characteristics and predicted ICI treatment responsivity of CRC patients in different MRS subgroups

**Figure 5:**
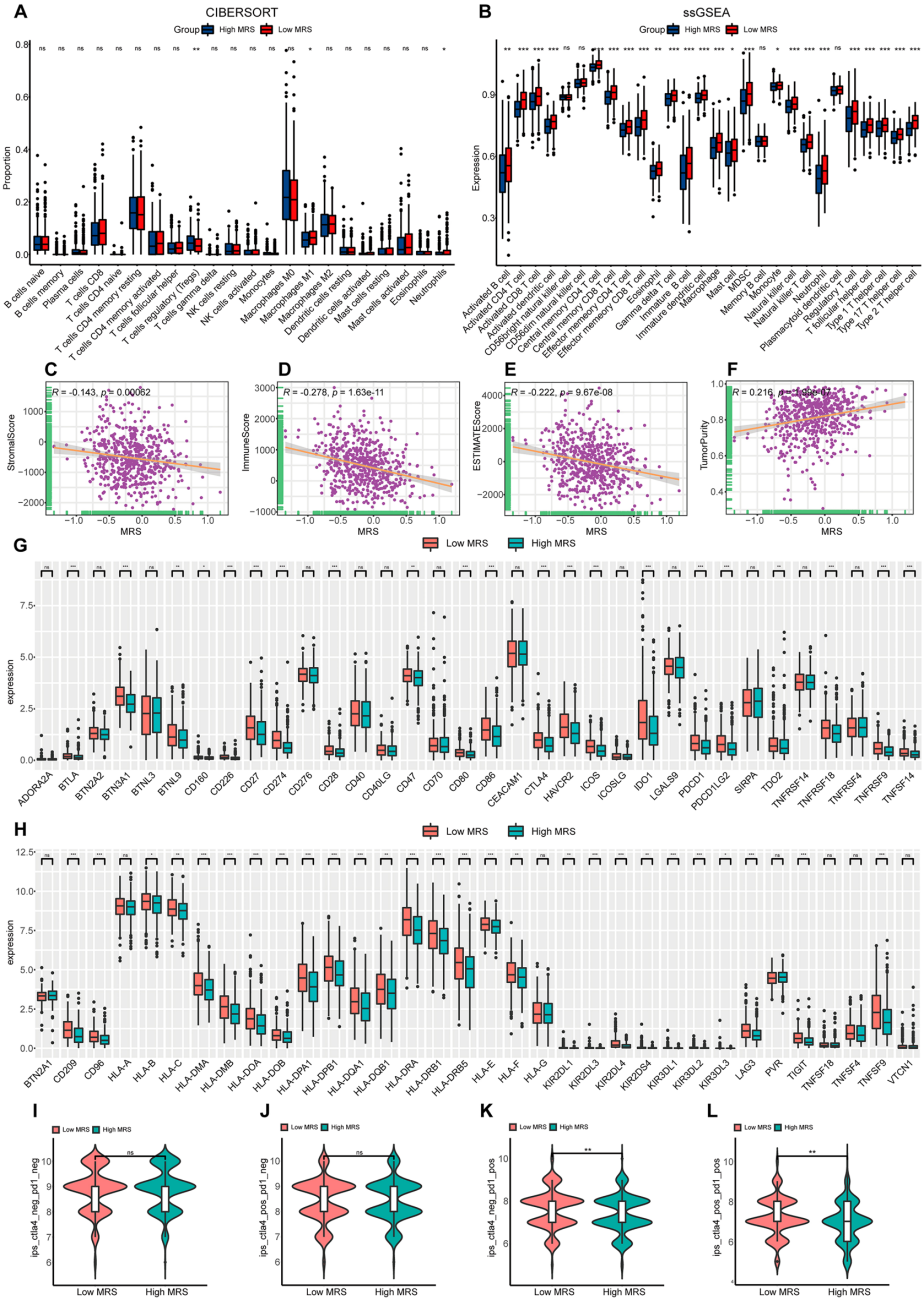
The immune characteristics of different MRS patient subgroups. (**A**) CIBERSORT analyses were used to assess immune cell infiltration in the indicated MRS subgroups. (**B**) ssGSEA analyses were used to assess immune cell-related gene expression in the indicated MRS subgroups. (**C**–**F**) Analyses of the relationship between MRS scores and stromal score (**C**), immune score (**D**), estimate score (**E**), and tumor purity (**F**). (**G**,**H**) The association between MRS subgroups and the expression of immune checkpoint genes. (**I**) The relationship between MRS subgroups and predicted immunotherapy outcomes. (**J**–**L**) The relationship between different MRS subgroups and predicted treatment outcomes for CTLA4 (**J**), PD-1 (**K**), or CTLA4 + PD-1 (**L**) targeted treatments. *P < 0.05; **P < 0.01; ***P < 0.001.

Given that the above GO and mutational analyses highlighted a potential relationship between MRS values and immune activity, the CIBERSORT algorithm and ssGSEA analyses were next used to more fully explore the immunological characteristics of patients in these two MRS subgroups. In the CIBERSORT analyses, patients in the low-MRS subgroup exhibited higher levels of M1 macrophage infiltration (Fig. 5A), and ssGSEA analyses suggested that low-MRS patient samples exhibited higher levels of infiltration by immune cell types including both activated CD4+ and CD8+ T cells (Fig. 5B). MRS scores were negatively correlated with stromal score, immune score, and assessment score values whereas they were positively correlated with tumor purity (Fig. 5C–F). While there were no significant differences in stromal scores between the two MRS patient subgroups, both immune scores and assessment scores were elevated in the low-MRS patient subgroup relative to the high-MRS subgroup, whereas tumor purity exhibited the opposite trend (Supplementary Fig. 2A–D). Next, the expression of 68 different immune checkpoint genes was assessed, revealing 48 to be differentially expressed between these two patient subgroups, with the majority of these genes, including PD-1 and CTLA4, being expressed at higher levels in the low-MRS group relative to the high-MRS group (Fig. 5G,H). The immunophenoscore (IPS) can be used to predict patient immunotherapy responsiveness^23^, and CRC patients in the low-MRS group exhibited significantly better IPS scores for both PD-1 and combination PD-1 + CTLA4 targeted immunotherapeutic treatment (Fig. 5I–L).

### WGCNA-based identification of CD8+ T cell-related hub genes

CD8+ T cells play a central role in tumor immunosurveillance, as they can detect neoantigens expressed by these malignancies and subsequently kill these tumor cells. Tumors, however, are able to evade these mechanisms by upregulating immune checkpoint proteins such as PD-L1, which can interact with PD-1 on the surface of CD8+ T cells and thereby suppress their activation and cytotoxicity. A WGCNA analysis was thus next conducted based on the genes that were differentially expressed in the two MRS patient subgroups (Supplementary Fig. 3A–D). In the resultant correlation heatmap, the identified turquoise module was found to be positively correlated with CD8+ T cells and activated CD8+ T cells, but negatively correlated with the progression of CRC (Supplementary Fig. 3E). Scatter plots highlighting the relationship between this turquoise module and key genes associated with CD8+ T cells and activated CD8+ T cells are provided in Supplementary Fig. 3F,G. In total, this turquoise module was comprised of 25 hub genes (AIM2, LY6G6F-LY6G6D, CD109, CIITA, CXCL10, CXCL11, CXCL5, FCGR3B, GBP4, GBP5, HMSD, IDO1, IFNG, KIR2DL4, KLRC4, KRT2, LY6G6D, M1AP, NCR1, PRDM8, RAB27B, TNNC2, TRIM7, TRPV6, ZNF683).

### Analyses of hub gene immune-related characteristics

**Figure 6:**
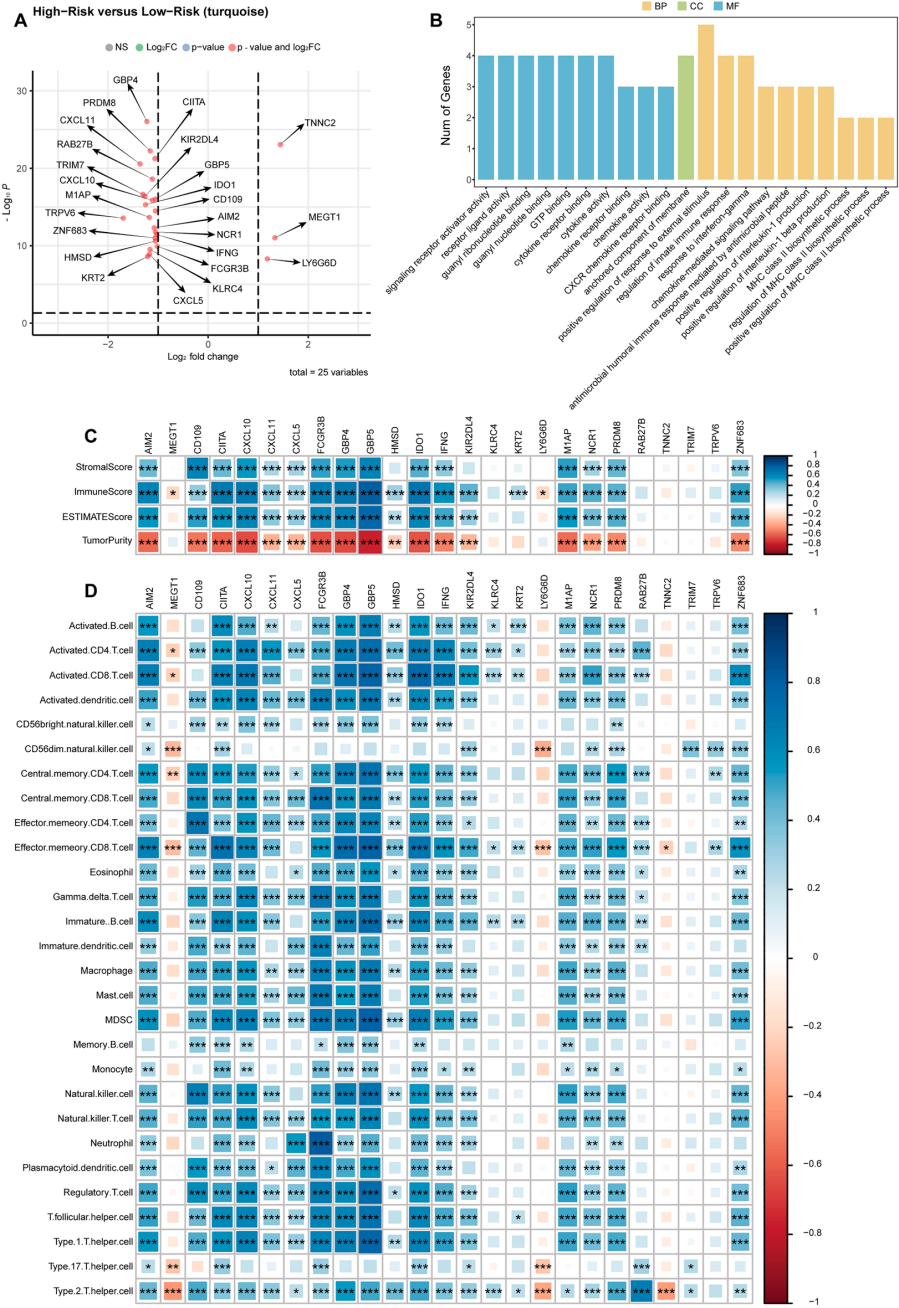
Analysis of hub gene immune-related characteristics. (**A**) Hub gene volcano plots. (**B**) Hub gene enrichment analyses. (**C**) The correlation heatmap between hub genes and industrial score, immune score, estimate score, and tumor purity is drawn by the corrplot function in the corrplot package of R language (version 4.1.2). (**D**) The heatmap of the correlation between the Hub gene and the immune cell expression of ssGSEA is drawn through the corrplot function of the corrplot package of R language (version 4.1.2). *P < 0.05; **P < 0.01; ***P < 0.001.

When comparing the expression of these 25 hub genes between the high- and low-MRS subgroups, 3 and 22 were respectively upregulated and downregulated (Fig. 6A). GO analyses indicated that these genes were associated with key immunological pathways including the chemokine-mediated signaling, chemokine receptor binding, and regulation of innate immune response pathways (Fig. 6B). Correlations between the expression of these 25 genes and stromal score, immune score, assessment score, and tumor score values in samples from patients in the high-MRS subgroup were assessed (Fig. 6C), as were correlations with immune cell-related gene expression (Fig. 6D).

### qRT-PCR validation of m^7^G-related gene expression

**Figure 7:**
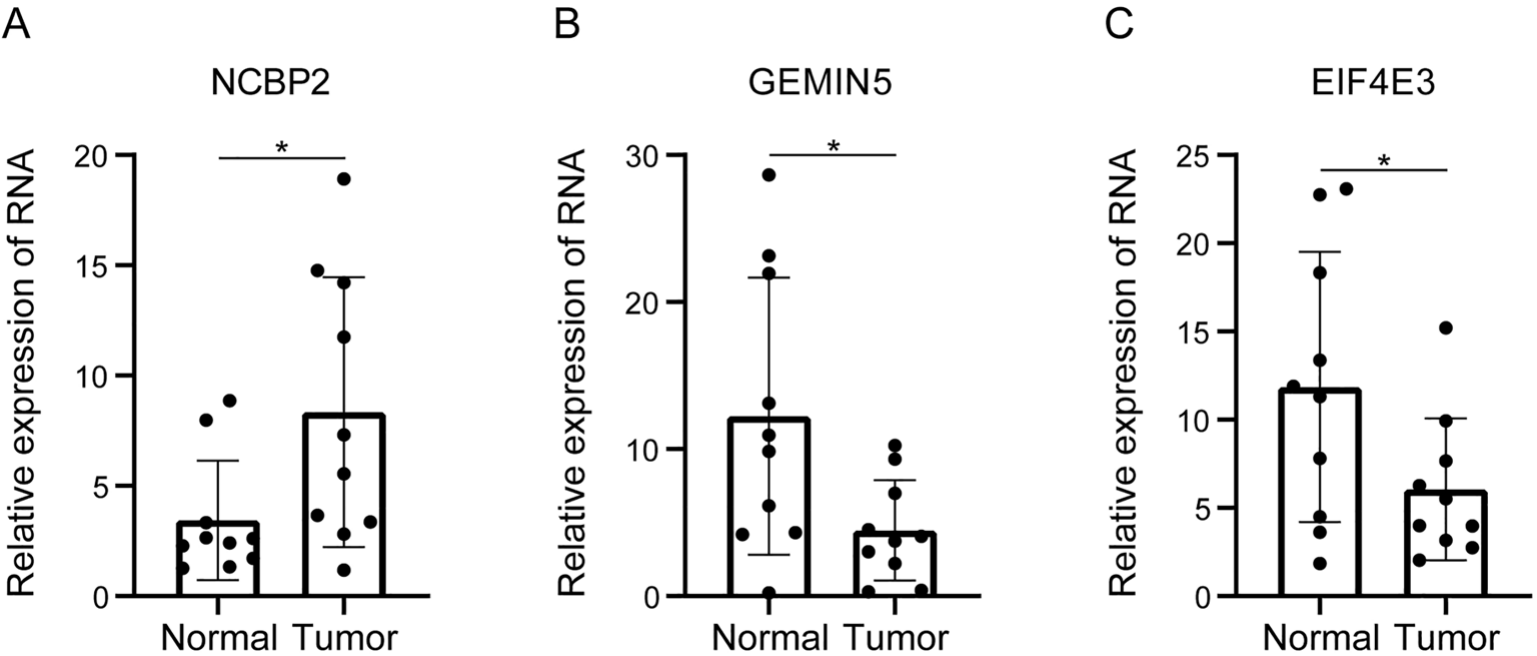
qRT-PCR validation of m^7^G-related gene expression. (**A**–**C**) qRT-PCR analysis of NCBP2, GEMIN5, and EIF4E3 expression in 10 groups of CRC tissues and paraneoplastic tissues. *P < 0.05; **P < 0.01; ***P < 0.001.

Lastly, 10 paired CRC tumor and paracancerous tissue samples were obtained, and qRT-PCR analyses revealed that EIF4E3 and GEMIN5 were downregulated in CRC, whereas NCBP2 was upregulated (Fig. 7A–C).

## Discussion

Over 170 different chemical RNA modifications have been identified to date and shown to influence cellular growth and other key physiological processes^13,14^. The m^7^G modification of tRNA, rRNA, mRNA 5′ cap, and mRNA internal regions is a relatively common finding in cells^15^, and such m^7^G modifications have recently been linked to the development and progression of tumors. METTL1, for example, can promote bladder cancer development^18^. The upregulation of METTL1 and WBSCR22 in glioma is also linked with the oncogenic process^21^, while METTL1 can inhibit PTEN signaling in hepatocellular carcinoma cells, thus enhancing their proliferative and migratory activity^24^. The present results further indicate that m^7^G-associated gene expression is closely linked to CRC patient prognosis and the immunogenicity of CRC tumors.

Initially, the mutational characteristics of 27 m^7^G-associated genes in CRC patient tumor tissues were analyzed, revealing mutations in 23 of these genes. However, these mutations were unrelated to CRC patient OS. The MRS model was then constructed via a LASSO regression approach based on m^7^G-associated EIF4E3, GEMIN5, and NCBP2 expression. In a prior report, EIF4E3 was identified as a novel m^7^G-associated tumor suppressor gene^25,26^, while GEMIN5 is an m^7^G cap-binding protein with unknown relevance in cancer^27^. Moreover, the m^7^G-associated gene NCBP2 has been linked to hepatocellular carcinoma patient prognostic outcomes. In Kaplan–Meier analyses, the OS of patients in the high-MRS subgroup was found to be worse than for low-MRS patients. MRS model validation was then performed with ROC curves and nomogram analyses, verifying the ability of MRS scores to predict CRC patient prognosis. Consistently, MRS scores were identified as an independent predictor of CRC patient OS. Moreover, in time-ROC analyses the area under the curve values at 3, 4 and 5 years were > 0.6, indicating that this model was capable of reliably gauging CRC patient survival outcomes in line with the results of nomogram-based analyses.

To investigate the ability of m^7^G-associated genes to influence the progression of CRC, genes that were differentially expressed in different MRS subgroups were identified and found to be closely associated with the CXCR chemokine receptor binding, humoral immune response, and antimicrobial humoral immune pathways. Analyses of the mutational landscapes in these two MRS subgroups further revealed that while APC mutation frequencies were similar in both groups, TP53 was more often mutated in the high-MRS group relative to the low-MRS group (49% vs. 69%). Tumors harboring such TP53 mutations tend to be more aggressive and associated with poorer prognostic outcomes^28,29^, particularly for CRC patients^30^. Prospective analyses of lung cancer patients have revealed that individuals harboring TP53 or KRAS mutations, and especially patients harboring mutations in both of these genes, can attain significant benefits from PD-1 inhibitor treatment^31^. Mutations in MUC16 are also related to better prognostic outcomes and a higher TMB in gastric cancer^32^, while mutations in TTN are linked to better ICI treatment outcomes in various solid tumors^33^. Thus, patients in the low-MRS subgroup may be more likely to respond well to immunotherapeutic treatment regimens. Several prospective clinical trials, including some conducted in CRC patients, have demonstrated the value of TMB as a biomarker capable of predicting ICI treatment responses. Higher TMB levels are also associated with prolonged OS following immunotherapy in most cancers^34–36^. Here, patients in the low-MRS subgroup exhibited a higher TMB than patients in the high-MRS subgroup. MSI refers to the deletion or insertion of repetitive units, with new microsatellite alleles appearing in tumors at specific loci that can function as biomarkers for PD-1 blockade^37^. The composition of the TME in CRC patients can be influenced by MSI status, thereby impacting ICI efficacy in these patients, with MSI-H patients being more likely to benefit from ICI treatment relative to MSI-L/MSS patients^38^. Consistently, lower MRS scores were observed for patients in the MSI-H group as compared to the MSI-L and MSS subgroups in this study, in line with the ability of low-MRS patients to benefit from ICI treatment. The loss of MMR function results in higher levels of DNA replication errors that are not properly repaired, increasing rates of somatic mutation^9,39^. Here, lower MRS scores were observed for patients harboring mutations in four MMR-related genes (MLH1, MSH2, MSH6, and PMS2), consistent with the lower MRS scores observed among MSI-H patients.

To better examine the utility of MRS scores as a biomarker capable of guiding patient immunotherapeutic treatment, further analyses of the TME in the high- and low-MRS subgroups were conducted. This approach revealed clear differences in immune cell composition within the TME of patients in these two subgroups, with higher levels of M1 macrophage infiltration and activated CD8+ T cell infiltration in the low-MRS group. This is important, given that CD8+ T cell infiltration is associated with better prognostic outcomes in many human cancers^40,41^. High levels of M1 macrophage infiltration are also associated with a more favorable prognosis in a range of malignancies^41–44^. When 68 different immune checkpoint genes were analyzed in these two MRS subgroups, 48 were found to be differentially expressed of which the majority were upregulated in low-MRS patients as compared to high-MRS patients. Immunotherapeutic regimens targeting PD-1 or CTLA4 have shown promise in the treatment of CRC patients^11,45,46^. Accordingly, the ability of this MRS model to predict patient responses to anti-PD-1/PD-L1 therapy was assessed by computing immunophenotype scores (IPS) values, revealing that individuals in the low-MRS group were more likely to respond to treatments targeting PD-1 or PD-1 + CTLA4. This suggests that treatment with ICIs is more likely to succeed in low-MRS patients. As such, these findings may provide a new foundation for the treatment of CRC patients^47^.

Lastly, 25 key CD8+ T cell infiltration-related hub genes were identified among the genes differentially expressed in different MRS patient subgroups (AIM2, LY6G6F-LY6G6D, CD109, CIITA, CXCL10, CXCL11, CXCL5, FCGR3B, GBP4, GBP5, HMSD, IDO1, (IFNG, KIR2DL4, KLRC4, KRT2, LY6G6D, M1AP, NCR1, PRDM8, RAB27B, TNNC2, TRIM7, TRPV6, ZNF683). The majority of these genes were downregulated in high-MRS patients, and correlation analyses confirmed that most of these genes were positively correlated with immune infiltration, in line with their downregulation in the high-MRS group. These differences may be related to patterns of m^7^G modification mediated by EIF4E3, GEMIN5, and NCBP2. Importantly, qRT-PCR analyses confirmed that EIF4E3 and GEMIN5 were downregulated in CRC patient samples, whereas NCBP2 was upregulated in these samples.

In conclusion, the MRS model developed in this study is a valuable biosignature that can aid in the prognostic classification of CRC patients and can also predict their likelihood of responding to ICI treatment. Despite these promising results, this study is subject to certain limitations. For one, the sample size for these analyses was limited. In addition, no validation of predicted ICI responsivity was conducted in an independent cohort of treated patients, highlighting an essential direction for subsequent studies. Further large-scale clinical trials will therefore be necessary to further confirm the clinical utility of this MRS model and to expand on the present results.

## Materials and methods

### Patient data collection

The GSE28722 dataset was downloaded from the GEO database (https://www.ncbi.nlm.nih.gov/geo/) and used as a validation dataset. The log2(x + 1) transformed TCGA-COAD dataset and the corresponding patient clinical information were downloaded from the UCSC Xena database (https://xenabrowser.net/datapages/). This dataset included 471 tumor samples and 41 non-tumor normal samples, with the TCGA-READ dataset (167 tumor samples and, normal non-tumor samples) being used for further analyses. Of the patients in these datasets, those with no clinical follow-up information or with an unknown survival time/survival status were excluded, with 567 CRC patients being included in the final study.

### Differentially expressed gene identification

The DESeq2 package and the R software environment (v 4.1.2) were used to identify genes that were differentially expressed in the TCGA patient cohort, with significant differentially expressed genes (DEGs) being identified with the following criteria: corrected adj.P < 0.05, |log2 FC|> 1.

### MRS model construction

Those m^7^G-associated genes exhibiting |LogFC|> 0.6 and P < 0.05 in analyses of the TCGA patient cohort were analyzed via univariate Cox regression analyses, with those genes attaining a P < 0.1 in these analyses being used to conduct LASSO analyses aimed at defining a risk-related prognostic m^7^G-associated gene (MRS) model as follows: MRS = EIF4E3 × (− 0.322311542390686) + GEMIN5 × (− 0.700547576450906) + NCBP2 × (0.506142039510906).

### Validation of the prognostic value of the MRS model

The prognostic utility of the established MRS model was assessed through Kaplan–Meier curve and ROC curve analyses^48^. Nomogram were used to assess the risk associated with 1-, 3-, and 5-year overall survival (OS)^49^, and the independent prognostic utility of MRS score values was assessed through univariate and multivariate Cox analyses.

### Mutational analyses

Somatic mutational data for 544 CRC patients were downloaded from the TCGA database. Samples not included in the present study were excluded from analysis, while the remaining 458 samples, which included 222 and 236 in the high- and low-MRS groups, respectively, were analyzed with the maftools package to assess mutational patterns. Then, tumor mutational burden (TMB) was calculated and a tumor mutation gene correlation heatmap was generated^50^.

### Immune cell infiltration analyses

Immune, stromal, estimated, and tumor purity scores for tumor samples were computed using appropriate R packages. The R GSVA package was used to conduct ssGSEA analyses designed to determine whether there were differences in immune cell infiltration of immunological function among different patient subgroups^51^.

### Heatmap

Mutation mapping correlation heat maps were drawn using the somatic interactions function of the maftools package. The correlation heat map was plotted using the corrplot function of the corrplot package. The gene expression heat map is plotted using the pheatmap function of the pheatmap package. All the above steps were done using the R language software of version 4.1.2.

### Patient samples

In total, 10 paired fresh CRC patient tumor and paracancerous tissue samples were obtained from the Affiliated Hospital of Nantong University. Patients had not undergone radiotherapeutic, chemotherapeutic, or immunotherapeutic treatment prior to sample collection. The ethics committee of the Affiliated Hospital of Nantong University approved this study, and all patients provided written informed consent.

### qRT-PCR

RNA was extracted using TRIzol (Invitrogen, USA). cDNA was generated in a volume of 20 µl using HiScript III RT SuperMix for qPCR (+ gDNA wiper) (Vazyme, Nanjing) according to the manufacturer's instructions. RT-qPCR analysis was then performed on a QuantStudio5 Real-Time PCR system (ABI, USA) using ChamQ Universal SYBR qPCR Master Mix (Vazyme, NJ). The primer sequences are as follows, GAPDH Forward: TGCACCACAACTGCTTAGC; GAPDH Reverse: GGCATGGACTGTGGTCATGAG; EIF4E3 Forward: AAGACTTGCCGAAGCCGATGC; EIF4E3 Reverse: ACCTGCCACTTTGAGTCCTAATTGC; GEMIN5 Forward: TAACAGAAATGACAGCCAGCACCTC; GEMIN5 Reverse: CACCACTATGCCATCCTTGTAGCC; NCBP2 Forward: GATGCTGGGAGAGGAGGCTATGG; NCBP2 Reverse: AATGGGCTCGTGTGCAGACTTTAG. All the above experiments were repeated three times.

### Statistical analysis

All statistical analyses were performed using R v 4.1.2 (https://www.r-project.org/) and GraphPad Prism 7, and P < 0.05 was the significance threshold.

## Supplementary Information

Supplementary Figures.

## Data Availability

CRC expression matrix data were obtained from TCGA database (https://portal.gdc.cancer.gov/) and GSE28722 dataset in GEO database (https://www.ncbi.nlm.nih.gov/geo/query/acc.cgi?a cc = GSE28722). Mutation spectrum data from TCGA database (https://portal.gdc.cancer.gov/). The above data sets are publicly available and can be downloaded from the Internet or obtained by contacting the authors.
