# Supplementary material for: The prognostic index of m7G-related genes in CRC correlates with immune infiltration
Source: Sci Rep. 2022 Dec 8;12:21282. doi: 10.1038/s41598-022-25823-w (PMC9732290; doi:10.1038/s41598-022-25823-w)
Supplement: Supplementary file 1 — Supplementary Figures. [file 41598_2022_25823_MOESM1_ESM.docx]

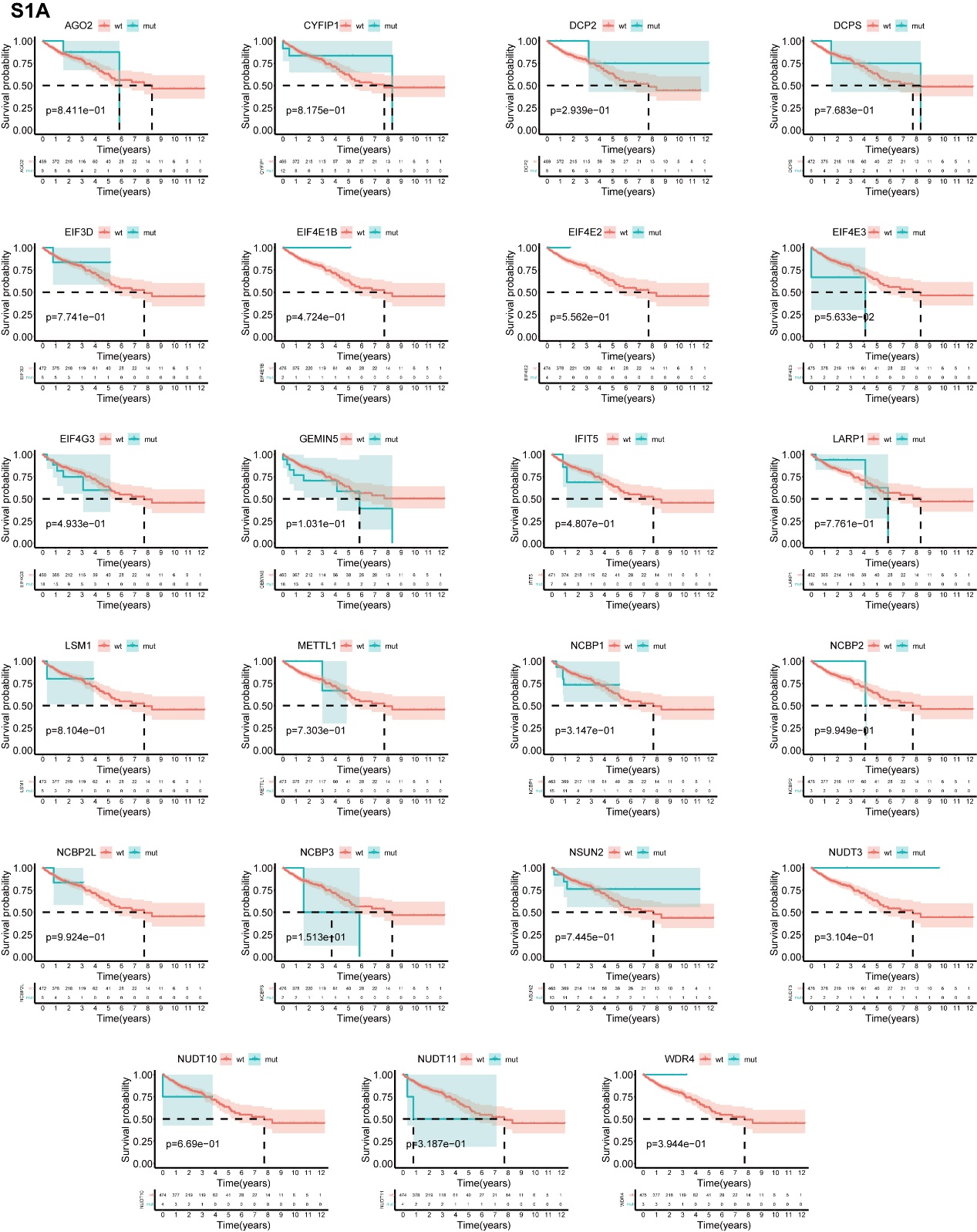


**Supplementary Figure 1 | Survival curve analysis of mutated and unmutated m7G-related genes.** (A) Mutation-associated Kaplan-Meier curve analysis of 23 m7G genes with mutations in CRC tumors.


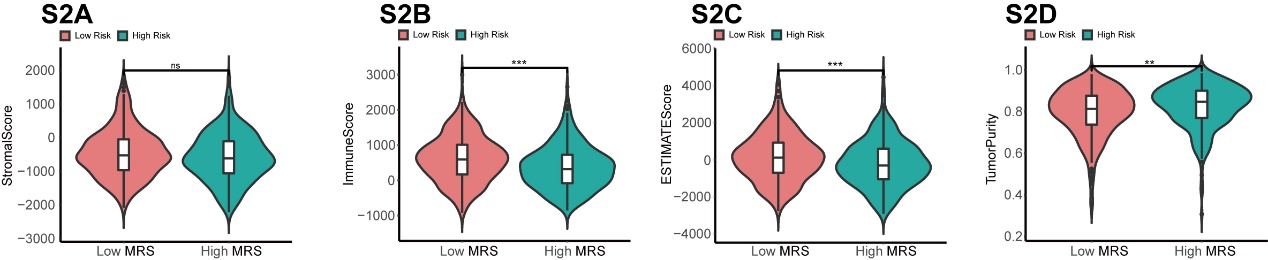


**Supplementary Figure 2 | Immune scores of different MRS Subgroups.** The relationship between different MRS subgroups and stromal score (A), immune score (B), estimate score (C), and tumor purity (D).


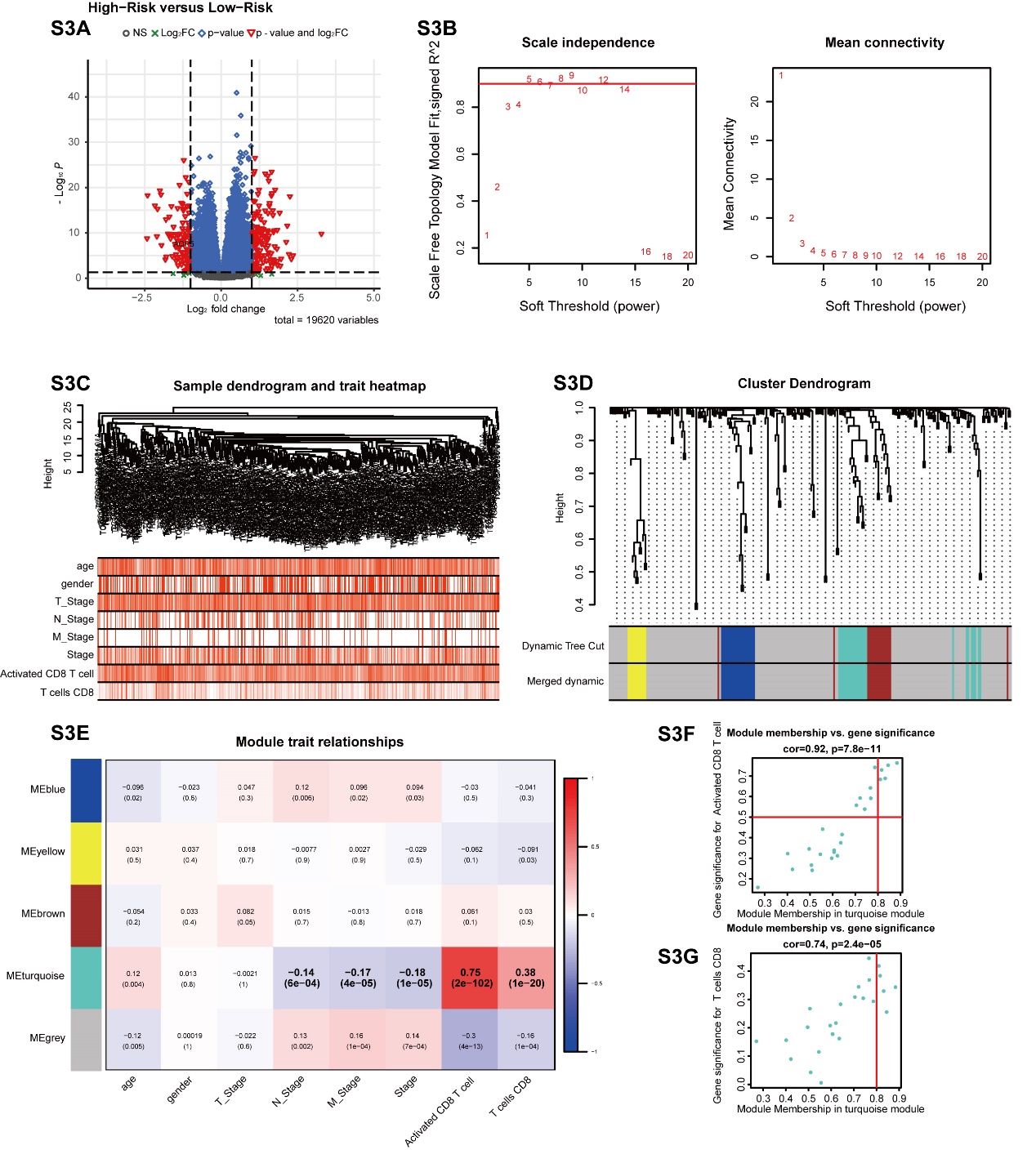


**Supplementary Figure 3 | WGCNA analysis to find immune-related Hub genes.** (A) Volcano map of differential genes between different MRS subgroups. (B-D) Dynamic cleavage of differential genes into 5 gene co-expression modules by WGCNA approach. (E) Correlation heatmap showing turquoise module correlates with N stage, M stage, pathological stage, and CD8+ T cell infiltration in CRC, visualized by the labeledHeatmap function of the WGCNA package in R language (Version 4.1.2). (F-G) Scatter plots of the module signature genes in turquoise modules.
